# Effect of non-pharmacological interventions on anxiety, depression, sleep quality, and pain after orthopedic surgery: A protocol for systematic review and network meta-analysis

**DOI:** 10.1097/MD.0000000000027645

**Published:** 2021-11-05

**Authors:** Xingquan Zhang, Genxin Zhou, Naifei Chen, Yonghua Zhang, Zenghui Gu

**Affiliations:** a Orthopedics and Traumatology of Traditional Chinese Medicine, Zhuji Sixth People's Hospital, Shaoxing Zhuji, Zhejiang, China; b Orthopedics and Traumatology of Traditional Chinese Medicine, Zhejiang Bone Injury Hospital, Hangzhou, Zhejiang, China; c Three Families of Bone, Sandun District of Zhejiang Hospital, Hangzhou, Zhejiang, China.

**Keywords:** network meta-analysis, non-pharmacological interventions, orthopedic surgery, protocol, systematic review

## Abstract

**Background:**

Patients after orthopedic surgery often experience the pain, anxiety, depression, and sleep disturbances, which can be greatly reduced by non-pharmacologic interventions as alternative therapies. Randomized controlled trials of nonpharmacologic interventions for anxiety, depression, sleep quality, and pain in patients after orthopedic surgery have been reported, but the results may be conflicting. Evidence to determine the optimal non-pharmacological intervention with a high efficacy is limited. This study aims to assess the effects of non-pharmacologic interventions on the bone anxiety, depression, sleep quality, and pain in patients after orthopedic surgery through a network meta-analysis, thus providing guidance in clinical application.

**Methods:**

A systematic search of randomized controlled trials reporting the effects of non-pharmacological interventions on anxiety, depression, sleep quality and pain after orthopedic surgery published before October 2021 will be searched in Wanfang, VIP Information Chinese Journal Service Platform, China National Knowledge Infrastructure, Chinese BioMedicine Literature Database, Pubmed, Embase, Cochrane, and Web of science. Two reviewers will be independently responsible for study selection, quality appraisal, and data extraction. Stata 14.0 software will be used to perform the network meta-analysis.

**Results:**

The findings of this research will be reported in a recognized journal.

**Conclusion:**

This meta-analysis will provide the stronger evidence for non-pharmacological interventions on alleviating bone anxiety, depression, sleep quality, and pain in patients after orthopedic surgery, which will help clinicians and decision makers in their choices.

Open Science Framework registration number: DOI 10.17605/OSF.IO/2SCBD.

## 1 Introduction

Patients after orthopedic surgery often experience the pain, anxiety, depression, and sleep disturbance problems.^[1,2]^ It is reported that orthopedic surgeries like those for joint replacements, extremity fractures, and spinal fusions are associated with higher levels of pain.^[3,4]^ Pain can cause negative emotions, such as anxiety, depression, insomnia, and unpleasantness, leading to tachycardia and elevated blood pressure in patients.^[5,6]^ Pain can lead to cardiovascular, neuroendocrine, gastrointestinal, and other system abnormalities. In addition, it also triggers emotional symptoms like the anxiety and insomnia, which can even aggravate the development of the disease and affect the postoperative recovery.^[7–9]^ Therefore, how to alleviate the pain, adverse emotions and psychological pressure, as well as improve the sleep quality is of significance to achieve a good outcome in postoperative orthopedic patients.

Currently, conventional clinical interventions include pharmacological and non-pharmacological interventions.^[10,11]^ Compared with pharmacological interventions, non-pharmacological interventions do not increase medical costs, treatment duration, or potential adverse effects.^[12]^ Non-pharmacologic interventions would not induce drug tolerance or dependence.^[13]^ Therefore, non-pharmacological interventions are often applied based on clinical need. It is shown that non-pharmacological interventions can effectively relieve cancer pain and chronic pain, regulate pain-induced negative emotions like the anxiety and depression, and promote the quality of sleep.^[14–16]^ Currently, non-pharmacological interventions have been widely used in patients with anxiety, depression, pain, and sleep disorders, which have achieved good outcomes.^[17–22]^

In postoperative orthopedic patients, non-pharmacological interventions have been validated effective in improving the quality of sleep, reducing pain, and relieving dysphoria.^[23–26]^ However, the difference in the efficacy of various non-pharmacological interventions on alleviating the anxiety, depression, sleep disorder, and pain in postoperative orthopedic surgery is unclear, which requires further explorations.

Network meta-analysis (NMA) is advanced from the traditional meta-analysis, which extends from a direct meta-analysis involving 2-group comparison to a simultaneous comparison of a series of treatment factors with each other.^[27]^ NMA is superior in quantitative analysis and comparison of summarizing different interventions for similar diseases, and ranking the effect of a certain indicator, thus selecting the optimal intervention.^[28]^ Therefore, NMA will be performed in the present study to evaluate the effects of different non-pharmacological interventions on the anxiety, depression, sleep quality, and pain after orthopedic surgery, thus providing guidance for clinical application.

## 2 Methods

### 2.1 Design and registration

This systematic review and network meta-analysis will evaluate the effectiveness and safety of non-pharmacological interventions on the anxiety, depression, sleep quality, and pain after orthopedic surgery. This protocol is designed according to the guideline of preferred reporting items for systematic review and meta-analysis protocols.^[29]^ The protocol of this review was registered in Open Science Framework (registration number: DOI 10.17605/OSF.IO/2SCBD). The findings of this study will be reported in line with the guideline of preferred reporting items for systematic reviews and network meta-analysis.^[30]^

### 2.2 Ethics

Since the present study is a meta-analysis based on existed researches, formal ethical approval is not required.

### 2.3 Eligibility criteria

#### 2.3.1 Type of studies

All randomized controlled trials (RCTs) reporting non-pharmacological interventions on the anxiety, depression, sleep quality, and pain after orthopedic surgery will be included in this study, regardless of blinding method.

#### 2.3.2 Type of participants

Postoperative orthopedic patients with 19 years of age and older.

#### 2.3.3 Type of interventions and comparators

RCTs evaluating the effectiveness of non-pharmacological on the anxiety, depression, sleep quality, and pain after orthopedic surgery.

Non-pharmacological interventions include psychotherapies, music therapy auricular therapy, acupressure, acupuncture therapy, and physical therapy. Studies combining non-pharmacological treatments and pharmacological treatments will be excluded, whereas those with a concomitant use of different non-pharmacological interventions will be included.

The control group will include conventional care, drug therapy, and placebo treatment.

#### 2.3.4 Outcome measures

Any rating scale that describes the anxiety, depression, sleep quality, and pain.

- Anxiety, including the Self-Rating Anxiety Scale (SAS) and Hamilton Anxiety Scale (HAMA);
- Depression, including the Geriatric Depression Scale (GDS), Hamilton Depression Rating Scale (HAMD), Beck Depression Inventory (BDI), Self-Rating Depression Scale (SDS), Center for Epidemiologic Studies Depression Scale (CES-D), and Edinburg Postpartum Depression Scale (EPDS);
- Sleep, including the Pittsburgh sleep quality index (PSQI), Self-Rating Scale of Sleep (SRSS), Dysfunctional Beliefs and attitudes about Sleep (DBAS), Ford Insomnia Response to Stress Test (FIRST), Insomnia Severity Index (ISI), and Sleep Dysfunction Rating Scale (SDRS);
- Pain, including the Visual Analogue Scale (VAS) and Numeric Rating Scales (NRS).

#### 2.3.5 Exclusion criteria

We will exclude non-RCT, RCTs without available data and duplicate publication.

### 2.4 Search strategy

We will systematically search RCTs published in English and Chinese language before October 2021 in Wanfang, VIP Information Chinese Journal Service Platform, China National Knowledge Infrastructure, Chinese BioMedicine Literature Database, Pubmed, Embase, Cochrane, and Web of science. In addition, we will also manually review citations in included RCTs. The preliminary search strategy of PubMed was shown in Table 1, which will be adjusted in accordance with specific database.

**Table 1:** Search strategy in PubMed database.

Number	Search terms
#1	Orthopedics[MeSH]
#2	Orthopedic Surgery[Title/Abstract]
#3	Surgery, Orthopedic[Title/Abstract]
#4	Orthopedic[Title/Abstract]
#5	Orthopedic Surgeries[Title/Abstract]
#6	Surgeries, Orthopedic[Title/Abstract]
#7	OR/1–6
#8	Anxiety[MeSH]
#9	Hypervigilance[Title/Abstract]
#10	Nervousness[Title/Abstract]
#11	Anxieties[Title/Abstract]
#12	Depression[MeSH]
#13	Depressive Symptoms[Title/Abstract]
#14	Emotional Depression[Title/Abstract]
#15	Depression, Emotional[Title/Abstract]
#16	Depressions[Title/Abstract]
#17	Depressions, Emotional[Title/Abstract]
#18	Depressive Symptom[Title/Abstract]
#19	Emotional Depressions[Title/Abstract]
#20	Symptom, Depressive[Title/Abstract]
#21	Symptoms, Depressive[Title/Abstract]
#22	Sleep[MeSH]
#23	Sleep, Slow-Wave[Title/Abstract]
#24	Sleep, Slow Wave[Title/Abstract]
#25	Slow-Wave Sleep[Title/Abstract]
#26	Pain[MeSH]
#27	Suffering, Physical[Title/Abstract]
#28	Ache[Title/Abstract]
#29	Pain, Burning[Title/Abstract]
#30	Pain, Crushing[Title/Abstract]
#31	Pain, Migratory[Title/Abstract]
#32	Pain, Radiating[Title/Abstract]
#33	Pain, Splitting[Title/Abstract]
#34	Aches[Title/Abstract]
#35	Burning Pain[Title/Abstract]
#36	Burning Pains[Title/Abstract]
#37	Crushing Pain[Title/Abstract]
#38	Crushing Pains[Title/Abstract]
#39	Migratory Pain[Title/Abstract]
#40	Migratory Pains[Title/Abstract]
#41	Pains, Burning[Title/Abstract]
#42	Pains, Crushing[Title/Abstract]
#43	Pains, Migratory[Title/Abstract]
#44	Pains, Radiating[Title/Abstract]
#45	Pains, Splitting[Title/Abstract]
#46	Physical Suffering[Title/Abstract]
#47	Physical Sufferings[Title/Abstract]
#48	Radiating Pain[Title/Abstract]
#49	Radiating Pains[Title/Abstract]
#50	Splitting Pain[Title/Abstract]
#51	Splitting Pains[Title/Abstract]
#52	Sufferings, Physical[Title/Abstract]
#53	OR/8-52
#54	Randomized Controlled Trials as Topic[MeSH]
#55	Clinical Trials, Randomized[Title/Abstract]
#56	Controlled Clinical Trials, Randomized[Title/Abstract]
#57	Trials, Randomized Clinical[Title/Abstract]
#58	Random∗[Title/Abstract]
#59	OR/54-58
#60	#7 AND #53 AND #59

### 2.5 Study selection and data extraction

**Figure 1:**
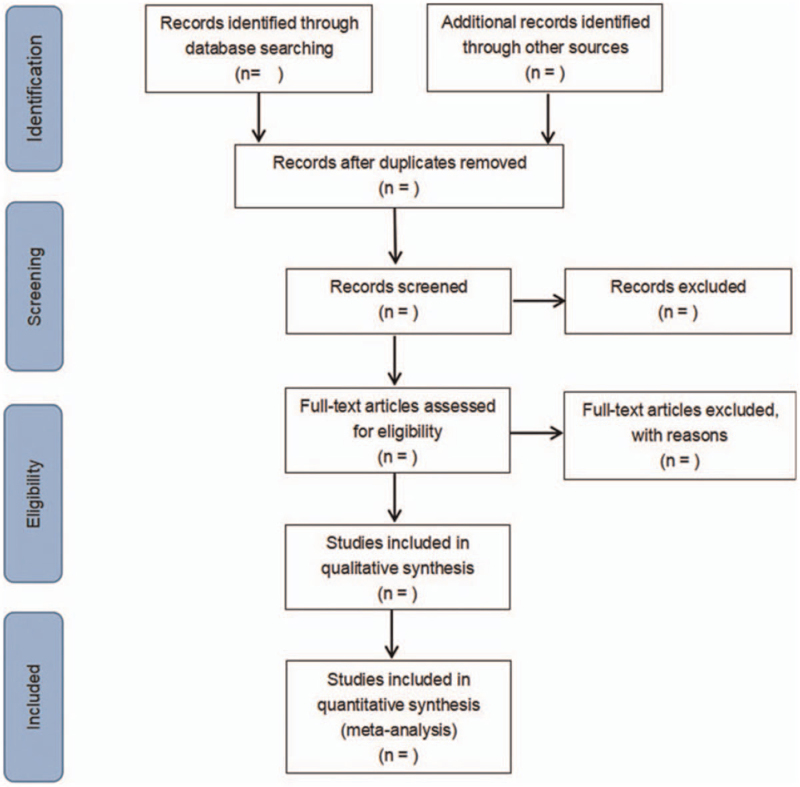
Flow diagram of study selection process.

All retrieved articles will be imported into Endnote. Duplicate RCTs will be deleted initially. Two researchers will be independently responsible for study screening and cross-check based on inclusion and exclusion criteria. For controversial literatures, consensus will be reached after discussion by a third researcher. Literature information of eligible RCTs will be extracted by 2 researchers. The following information will be extracted: Study characteristics: title, name of the first author, publication date, literature sources, and quality evaluation. Baseline characteristics of patients: sample size, age, gender, surgery type, and method, procedure, frequency and duration of interventions; Outcomes: Assessment scale and scores before and after intervention. Any disagreements will be resolved through discussion or consultation with a third researcher. The screening flow chart of this study was presented in Figure 1.

### 2.6 Quality assessment of studies

The quality of RCTs will be assessed using the Cochrane Collaboration risk assessment tool.^[31]^ The evaluation results will be classified into the high-risk, low-risk, and unclear categories.

### 2.7 Data synthesis and statistical methods

#### 2.7.1 Pairwise and network meta-analysis

We will conduct the conventional pairwise meta-analysis for direct comparisons. The effect size of continuous variable data will be calculated with the standardized mean difference (SMD) and corresponding 95% confidence intervals. Statistical analyses will be performed using Stata 14.0 software (STATA Corporation, College Station, TX). The network, mvmeta program package and funnel plots will be depicted to visualize the net relationship between the interventions for comparison, and assess publication bias, respectively. The surface under the cumulative ranking curve (SUCRA) and probability values will be summarized and reported as SUCRA for each non-pharmacological intervention. SUCRA curves will be described with percentages, with 100% for the best treatment and 0% for the worst.

#### 2.7.2 Assessment of heterogeneity

Before the combination of effect size, the homogeneity will be analyzed by *I*^2^ statistic to check whether the results of individual studies are mergeable. A fixed-effect model will be used to combine the effect size at *I*^2^ ≤ 50%; Otherwise, a random-effect model will be used for meta-analysis after excluding the influence of apparent clinical heterogeneity.

#### 2.7.3 Subgroup and sensitivity analysis

The subgroup analysis will be conducted based on different assessment tools and the surgery type. Sensitivity analysis will be performed by a one-by-one elimination method to verify the robustness of the results.

#### 2.7.4 Assessment of inconsistency

Consistency is a basic principle in network meta-analysis, indicating the consistency between direct and indirect comparative results. The node-splitting approach and inconsistency model will be used to test the consistency assumption.^[32]^

#### 2.7.5 Publication bias

Comparison-adjusted funnel plots will be drawn to evaluate publication bias.^[33]^

### 2.8 Grading the quality of evidence

The evidence quality will be evaluated by 2 independent reviewers using the Grading of Recommendations Assessment, Development and Evaluation (GRADE), which classifies the quality of evidence as high, medium, low, and very low.^[34]^

## 3 Discussion

Orthopedic patients often experience varying degrees of pain with the gradually regressive anesthesia. In addition, the postoperative pain after orthopedic surgery may also influence the quality of sleep, which in turn causes psychological stress and affects the recovery.^[17]^ Therefore, it is very important to relieve the pain to promote effective recovery of postoperative orthopedic patients.^[35]^

Western medical treatment commonly uses oral medication for analgesia, although it is featured by limited effects, and potential adverse events.^[36]^ Non-pharmacological interventions, including psychotherapy, music therapy, auricular therapy, acupressure, acupuncture therapy, and physical therapy are effective in alleviating pain and dysphoria.^[37–40]^ Many non-pharmacological interventions can be used to improve postoperative orthopedic anxiety, depression, sleep quality, and pain.^[23–26]^ However, previous studies have not elucidated which types of non-pharmacologic interventions are the most effective for postoperative orthopedic anxiety, depression, sleep quality, and pain. Therefore, this study aims to evaluate the effects of different non-pharmacological interventions on anxiety, depression, sleep quality, and pain after orthopedic surgery using the NMA method, thus guiding the clinical practice.

## Author contributions

**Conceptualization:** Xingquan Zhang.

**Data collection:** Genxin Zhou and Naifei Chen.

**Funding acquisition:** Xingquan Zhang.

**Resources:** Yonghua Zhang.

**Software:** Zenghui Gu.

**Supervision:** Xingquan Zhang.

**Writing – original draft:** Xingquan Zhang.

**Writing – review & editing:** Xingquan Zhang and Genxin Zhou.
